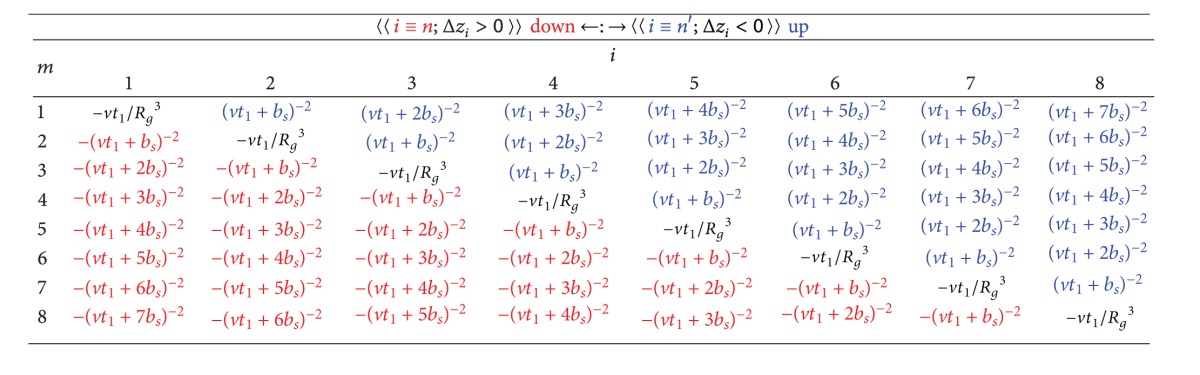# Translational Dielectric Friction on a Chain of Charged Spheres

**DOI:** 10.1155/2014/567560

**Published:** 2014-01-29

**Authors:** Sondès Boughammoura, Jalel M'halla

**Affiliations:** Faculty of Sciences of Monastir, UR Electrolytes, University of Monastir, 5000 Monastir, Tunisia

## Abstract

We have proved in details that the dielectric friction remains the principal frictional effect for a stretched polyion modeled as a chain of charged spheres, whereas, in the case of Manning's model (infinite thread with a continuous distribution of charge), this friction effect is nonexistent. According to this chain model, it is therefore possible to detect by conductivity measurements any transition from a coiled configuration (ellipsoidal model) to a stretched configuration during dilution process. We have also underlined the important interdependence between the dielectric friction and the ionic condensation of the counterions, in order to distinguish between the Ostwald regime and the Manning regime for which the degree of condensation is practically constant in a large range of concentrations.

## 1. Introduction

It was generally assumed that, when the concentration *C*_*P*_ of a polyelectrolyte is sufficiently low, screen effect due to free counterions of ionic atmosphere is relatively weak so that the intrarepulsion between charged monomers inside each flexible chain of structural length *L*_*S*_ prevents the collapse of the polyion and consequently the stretched configuration becomes the most probable at high dilution. This prevention is enhanced in the case of polyelectrolytes which present a concentration regime satisfying the Ostwald dilution principle, since in this case the degree of ionic condensation (1 − *α*) of counterions on charged monomers (partial neutralization of the structural charge *eZ*_*S*_ of the polyion) decreases with dilution [1–4]. It follows that continuous transition from stretched (or rod-like) conformation to coiled shape could be observed by increasing the concentration of counterions: *C*_*i*_ = (*Z*_*S*_/*Z*_*i*_)*C*_*P*_, or by decreasing the permittivity *ε* of the solvent. Indeed, since the concentration effect changes the apparent charge, *eZ*_app_ = *eαZ*_*S*_, the shape, and the size of polyions and since, in their turn, these parameters govern the different frictional processes, it therefore results in more or less sharp variation of the mobility of the polyions with the concentration. In previous studies [3, 5], the various friction effects on polyions have been classified to five types: (a) the hydrodynamic friction which depends on the viscosity of the solvent, *η*, the size and the shape of the polyion, and it is quantitatively evaluated by the value of the hydrodynamic equivalent conductivity of the polyion, *λ*_*P*_^°HD^; (b) the electrophoretic friction which expresses the hydrodynamic friction on the ionic atmosphere of the polyion, and it is quantified by the electrophoretic conductibility increment, Δ*λ*_*P*_^el^; (c) the ionic friction (or ionic relaxation effect) due to the local field Δ**X** ^ir^ caused by the polarization of the ionic atmosphere by the external field **E** during its relaxation so that the frictional force acting on the polyion is equal to Δ**F** ^ir^ = *eZ*_app_ · Δ**X** ^ir^, the intensity of this effect is evaluated by the ionic friction coefficient *β* ^ir^_*P*_ = |Δ**X** ^ir^/**E**|; (d) the translational dielectric friction effect [3, 6] due to the perturbation of the polarization of solvent molecules around the moving polyion. This effect is evaluated by the coefficient *β* ^df^_*P*_ = |Δ**Χ** ^dr^/**E**|, where Δ**Χ** ^dr^ is the local dielectric field. Note that *β* ^df^_*P*_ is proportional to the square power of the degree *α* of dissociation: *β* ^df^_*P*_ = *α* ^2^ *β*°_*P*_^df^.

The relative importance of each friction contribution depends on the concentration regime and on the conformation of the polyion. However, it is possible to express formally, in the general case, the equivalent conductivity of the polyion *λ*_*P*_ in terms of the different friction contributions as follows [3, 6]:

(1) $$\begin{array}{c} \lambda_{P}\approx {\left(1+\beta^{\text{ir}}\right)}^{-1}\left[\frac{\left(\alpha {\lambda_{P}}^{{}^{\circ}\text{HD}}-\Delta {\lambda_{P}}^{\text{el}}\right)}{\left(1+\alpha^{2}{{\beta^{{}^{\circ}}}_{P}}^{\text{df}}\right)}\right]. \end{array}$$

**Figure 1:**
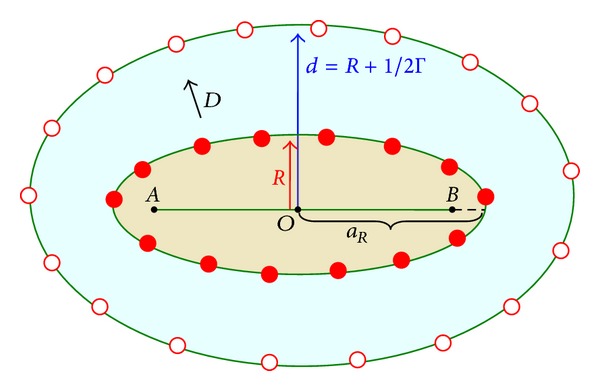
The polyion is equivalent to an ellipsoidal capacitor of (1/2Γ) thickness. 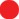 is a condensed counterion and 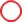 is a free counterion. **D** is the displacement field due to both the polyion and the condensed counterions.

For ellipsoidal polyions (see Figure 1) of focuses *A* and *B*, of interfocuses distance: *L* = *AB* ≤ *L*_*S*_, of minor axis *R*, and major axis *a*_*R*_ = (*R* ^2^ + *L* ^2^/4)^1/2^, obeying Ostwald regime, so that *α* → 1, when *C*_*P*_ → 0, we have generalized the Debye-Onsager theory concerning simple electrolytes for the calculation of the electrophoretic increment Δ*λ*_*P*_^el^ and the ionic friction coefficient *β* ^ir^ [6]. On the other hand, we have generalized the Boyd-Zwanzig's approach [7] concerning simple spherical ions, to the case of ellipsoidal polyions [6], in order to evaluate the dielectric friction coefficient at infinite dilution *β*°_*P*_^df^. The different contributions have been expressed in terms of the degree of dissociation of counterions *α*, the geometric parameters of the ellipsoidal polyion *R* and *L*, the effective radius of the counterions *R*_*k*_, the charge numbers of the polyion and the counterions respectively, *Z*_*S*_, *Z*_*k*_, and also in terms of the minor axis of the ellipsoidal ionic atmosphere surrounding the polyion, *d*, (see Figure 1) which depends on the concentration *αC*°_*k*_ of free counterions and therefore on the Debye-MSA [8–11] screen parameters Γ_MSA_ and *χ*_*D*_ (in Å^−1^):

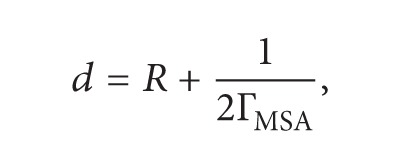
(2)

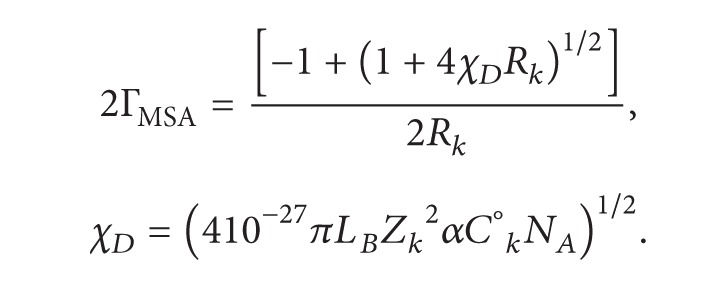
(3)
*N*_*A*_ is the Avogadro number and *L*_*B*_ = *e* ^2^/*εk*_*B*_ *T* is the Bjerrum length, so that the hydrodynamic and the electrophoretic contributions are respectively given by

(4) $$\begin{array}{c} {\lambda_{P}}^{{}^{\circ}\text{HD}}=\left(\frac{\left|Z_{S}\right|Fe}{6\pi \eta \langle R\rangle }\right), \end{array}$$

(5) $$\begin{array}{c} \Delta {\lambda^{\text{el}}}_{P}=\left(\frac{\alpha \left|Z_{S}\right|Fe}{6\pi \eta \langle d\rangle }\right). \end{array}$$

*F* = *eN*_*A*_ is the Faraday, 〈*R*〉 and 〈*d*〉 are, respectively, the mean radius of the polyion and of its ionic atmosphere defined as follows:

(6) $$\begin{array}{c} \langle R\rangle =\frac{L}{L_{n}\left[g\left(R,L\right)\right]};\;\;\langle d\rangle =\frac{L}{L_{n}\left[g\left(d,L\right)\right]}, \end{array}$$

where *g*(*x*, *L*) is the so-called “generating function,” characterizing the conformation of the polyion (and its ionic atmosphere) which can vary from the spherical shape (*L* = 0) to the cylindrical configuration (*L* = *L*_*S*_) [3, 6]:

(7) $$\begin{array}{c} g\left(x,L\right)=\frac{\left[{\left(4x^{2}+L^{2}\right)}^{1/2}+L\right]}{\left[{\left(4x^{2}+L^{2}\right)}^{1/2}-L\right]}. \end{array}$$

Note that 〈*R*〉 measures also the electrostatic capacitance *C*_ap_ of the ellipsoidal polyion in c.g.s.u.e units [6] and (*C*_ap_′)^−1^ = (〈*R*〉^−1^ − 〈*d*〉^−1^) measures also the inverse of the electrostatic capacitance *C*_ap_′ of the ellipsoidal capacitor constituted by the polyion and its ionic atmosphere “Gouy capacitance” (see Figure 1).

The ionic friction due to the perturbation of the ionic atmosphere during its relaxation is expressed in terms of the coefficient *β*_P_^ir^ as follows:

(8) $$\begin{array}{c} {\beta_{\text{P}}}^{\text{ir}}=\frac{\alpha \left|Z_{S}Z_{i}\right|L_{B}\left(3d^{2}+L^{2}/4\right)}{\left[18{\left(d^{2}+L^{2}/4\right)}^{3/2}\right]}. \end{array}$$

Note that in the limiting case of Manning's model (i.e., *L* → *∞*, *α* = *α*_*M*_ = *L*_*S*_/(|*Z*_*S*_ *Z*_*k*_ | *L*_*B*_)), the ionic friction coefficient *β*_P_^ir^ → (1/9).

It follows according to (2), (3), (5), and (8) that the contributions Δ*λ* ^el^_*P*_ and *β*_*P*_^ir^, relative to ellipsoidal polyions, vanish at infinite dilution *C*_*P*_ → 0, *α* → 1, *d* → *∞*, so that

(9) $$\begin{array}{c} \lambda_{P}⟶\frac{{\lambda_{P}}^{{}^{\circ}\text{HD}}}{\left(1+{{\beta^{{}^{\circ}}}_{P}}^{\text{df}}\right)}. \end{array}$$

The general expression of the dielectric friction coefficient *β*°_*P*_^df^ is [6]

(10) $$\begin{array}{c} {{\beta^{{}^{\circ}}}_{P}}^{\text{df}}=\left(\frac{2}{3}\right){\left|Z_{S}\right|}^{2}{\left(\frac{R_{w}}{R_{\text{app}}}\right)}^{3}\left(\frac{L_{B}}{\langle R\rangle }\right)\left[1-\frac{\varepsilon_{\infty }}{\varepsilon }\right]. \end{array}$$

*ε* and *ε*_*∞*_ are, respectively, the static and the high-frequency dielectric constants of the solvent. The apparent radius *R*_app_ is a function of the eccentricity of the polyion *γ* = *L*/2*R* so that *R*_app_ ≈ 〈*R*〉 for *γ* < 1 and *R*_app_ ≈ *L*/2 for *γ* > 1 [6]. For spherical polyion (*γ* = 0), *R*_app_ = *R*, and (10) becomes in this case identical to Boyd-Zwanzig's equation. Note that the dielectric friction coefficient at infinite dilution *β*°_*P*_^df^ is in general of the order of few percent and *decreases with elongation L* of the polyion so that the conductibility of the polyion is essentially governed by the ionic condensation process and the hydrodynamic friction *λ*_Poly_ ≈ *αλ*_*P*_^°HD^.

In the case of stretched polyions obeying Manning's regime (*R*/*L*) → 0, *β*°_*P*_^df^ is negligible, whereas *α* and *β*_P_^ir^ remain relatively important and especially quasi-independent on the concentration so that *λ*_*P*_ ≈ *αλ*_*P*_^°HD^(1+*β* ^ir^)^−1^[1 − (〈*R*〉/〈*d*〉)].

However, in a recent work we have observed a *sharp increase* of the dielectric friction due to the* elongation* of some polyions during the *dilution* process [12–16]. We have concluded that (10) predicting the *decrease* of the dielectric friction coefficient *β*°_*P*_^df^ with the elongation *L* is only valid for stretched polyions characterized by a *continuous* linear distribution of their apparent charge *eZ*_app_.

In fact, the real structure of a stretched polyion is rather similar to a chain of charged spheres than to a rod uniformly charged. In other terms, the stretched polyion could be modeled as a succession of |*Z*_*S*_| charged rigid spherical monomers “pearls” or “groups” of *R*_*g*_ radius and ±*e* charge. The charge distribution of the polyion is therefore discontinuous “necklace.” The distance separating two successive groups is equal to *b*_*S*_ = *L*_*S*_/|*Z*_*S*_ | ≥2*R*_*g*_. Note that this model converges toward the rod-like Manning's model when the ratio *L*_*S*_/*R*_*g*_ → *∞*. On the other hand, for coiled conformation, the polyion could be treated as a rigid ellipsoid uniformly charged of *A* and *B* focuses, *L* length, *R* minor axis, and (*Z*_*S*_ *e*) charge.

The essential difference between the chain configuration and the ellipsoidal conformation concerns the importance of the dielectric friction. In the first case, the total dielectric friction results from the superposition of the various dielectric frictions acting on the successive |*Z*_*S*_| charged groups so that the corresponding dielectric friction coefficient *β*°_*P*_^df^ is proportional to the ratio (|*Z*_*S*_ | *e* ^2^)/*R*_*g*_^3^, whereas, in the second case, the friction coefficient is proportional to (*eZ*_*S*_)^2^/*R*_app_^3^. Consequently, an important variation of the dielectric friction is expected during this conformation transition.

The main objective of the present work is therefore to explain in details why the transition from coiled configuration to completely stretched chain is accompanied by a sharp increase of the dielectric friction on the moving polyion, whereas such variation is undetectable according to the model of Manning.

In order to achieve this objective progressively and completely we have organized the rest of the paper as follows. In Section 2 we present an new simplified (heuristic) derivation of the expression of the dielectric frictional force **F** ^dr^ acting on a moving spherical charge, different from those of Zwanzig [7], Hubbard and Douglas [17], and Hubbard and Onsager [18] which has the advantage of underlining the physical significance of the process of dielectric friction. In Section 3 we give a brief recall of the general expression of **F** ^dr^ according to the time-correlation function formalism [6] in terms of the displacement field **D**(**r**′, *t*) created by the polyion, the memory function *ζ*(*t*) relating the polarization **P**(**r**′, *t*) around the polyion to **D**(**r**′, *t*), and the key integral *I*_*z*_(*t*) allowing the direct calculation of **F** ^dr^ via *ζ*(*t*) and **D**(**r**′, *t*). In Section 4 we apply the previous approach to calculate in details the explicit expression of *I*_*z*_(*t*) and therefore the frictional force **F** ^dr^ in the case of a chain of charged spheres. In Section 5 we discuss the coupling between the dielectric friction effect and the ionic condensation processes on the basis of a generalization of the Fuoss's approach.  Section 6 gives the explicit expression of the variation of the conductibility of a polyion with dilution.  Section 7 concludes with a brief discussion of the results using some experiments.

## 2. A Simplified Derivation of the Expression of the Dielectric Frictional Force on a Moving Charged Rigid Sphere

**Figure 2:**
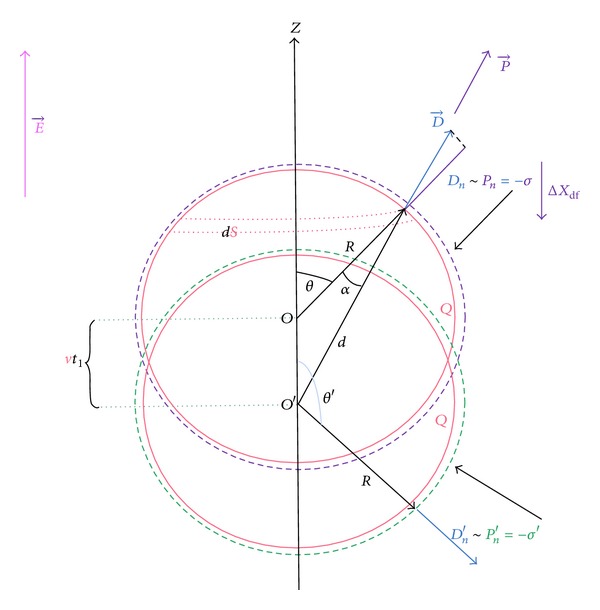
Representation of a moving spherical polyion at time *t* and time (*t* − *t*_1_).

Figure 2 represents a moving rigid sphere along the *z*-axis, of *R* radius, *Q* charge, and velocity *v*. *O* represents its center at time “*t*” and *O*′ its center at a previous instant (*t* − *t*_1_) so that the distance *OO*′ is equal to *vt*_1_. The moving charge *Q* creates at each point **r** of the dielectric medium, a time dependent electric displacement **D**(**r**, *t*) which in its turn polarizes the solvent molecules. We design by **P**(*R*, *θ*, *t*) the induced polarization at the surface of the sphere *O* and by **P**′(*R*, *θ*′, *t* − *t*_1_) the induced polarization at the surface of the sphere *O*′ at *t* − *t*_1_. Now, according to the theory of dielectric mediums, the superficial charge density *σ*(*R*, *θ*, *t*) of the dielectric molecules at the interface between the solvent and the charged sphere *O* at time *t* is related to the orthogonal component *P*_*n*_(*R*, *θ*, *t*) of the induced polarization **P**(*R*, *θ*, *t*) at the surface of the sphere *O* as follows:

(11) $$\begin{array}{c} \sigma \left(R,\theta ,t\right)=-P_{n}\left(R,\theta ,t\right). \end{array}$$

On the same way the charge density *σ*′(*R*, *θ*′, *t* − *t*_1_) is equal to

(12) $$\begin{array}{c} \sigma^{\prime }\left(R,\theta^{\prime },t-t_{1}\right)=-P_{n}^{\prime }\left(R,\theta^{\prime },t-t_{1}\right). \end{array}$$

On the other hand, according to time-correlation function formalism, *P*_*n*_(*R*, *θ*, *t*) can be expressed via a temporal convolution integral, as a sum of linear responses to the successive orthogonal components *D*_*n*_(*R*, *θ*, *t* − *t*_1_) of **D**(*R*, *θ*, *t* − *t*_1_) at the different anterior instants (*t* − *t*_1_):

(13) −σ(R,θ,t)=Pn(R,θ,t)=(14π)∫0∞ζ(t1)Dn(R,t−t1)dt1.

According to Figure 2,

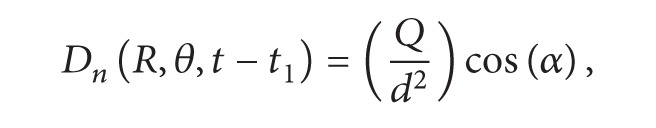
(14)

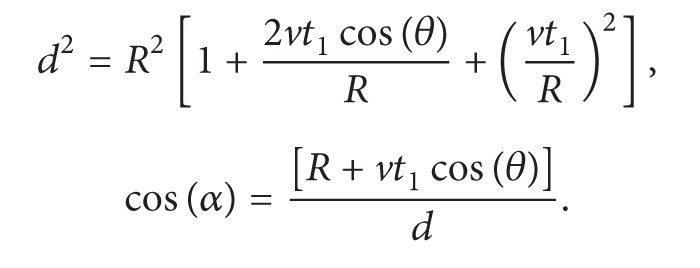
(15)
*ζ*(*t*) is the associated after-effect function representing both electronic relaxation and rotational diffusion of dipolar molecules [7]:

(16) ζ(t)=[1−(1ε∞)]δ(t)+[(ε−ε∞)(τε∞2)−1exp⁡(−εtε∞τ)].

*τ* is the relaxation time of the solvent molecules and *ε* and *ε*_*∞*_ are the static and high-frequency dielectric constants of the solvent. For water at 25°C, *ε* = 78.3 and *ε*_*∞*_ = 1.77. Notice that as the ratio *ε*/*ε*_*∞*_ ≫ 1, the exponential function exp(−*εt*/*ε*_*∞*_ *τ*) decreases sharply with time and therefore the temporal correlation between *P*_*n*_ and *D*_*n*_ vanishes rapidly when *t*_1_ → *τ*.

The expression of *σ*′(*R*, *θ*′, *t* − *t*_1_) is more ordinary because it is induced by the displacement field *D*_*n*_′(*R*) = (*Q*/*R* ^2^) which is constant for all *θ*′ so that

(17) $$\begin{array}{c} \sigma^{\prime }\left(R,\theta^{\prime },t-t_{1}\right)\sim -\left(\frac{Q}{4\pi R^{2}}\right). \end{array}$$

Slowly moving particle is characterized by the condition *vτ* < *R*, so that *vt*_1_ ≤ *R* during the period of correlation (*t*_1_ < *τ*). Now, according to (15), cos(*α*) is always > 0; it follows therefore from (13) that for *t*_1_ < *τ*, *σ*(*R*, *θ*, *t*)~−*Q* for all cos(*θ*). In the case of speedy particle so that *vt*_1_ > *R*, (13) and (14) imply that *σ*(*R*, *θ*, *t*)~−*Q* for all *θ* < *π*/2 and *σ*(*R*, *θ*, *t*)~+*Q* for all *θ* > *π*/2. Note also that *σ*′(*R*, *θ*′, *t* − *t*_1_)~−*Q* for all *θ*′.

In both cases, the dielectric superficial charges *σ*(*R*, *θ*, *t*) and *σ*′(*R*, *θ*′, *t* − *t*_1_) present an axial symmetry around the *z*-axis, and consequently they are created at the center *O* of the moving sphere, and, at time *t*, a reacting dielectric relaxation field Δ**Χ** ^dr^ is directed along the *z*-axis. According to the principle of superposition, its *z* component Δ*X*_*z*_^dr^ can be expressed as a sum of two local fields, respectively, Δ*X*_*σz*_^dr^ and Δ*X*_*σ*′*z*_^dr^:

(18) $$\begin{array}{c} \Delta {X_{z}}^{\text{dr}}=\Delta {X_{\sigma z}}^{\text{dr}}+\Delta {X_{\sigma^{\prime }z}}^{\text{dr}}. \end{array}$$

The relation between the *z* component Δ*X*_*σz*_^dr^ and *σ*(*R*, *θ*, *t*) is obviously

(19) $$\begin{array}{c} \Delta {X_{\sigma z}}^{\text{dr}}=-\left(\frac{1}{R^{2}}\right)\overset{}{\underset{}{\int }}\left[\sigma \left(R,\theta ,t\right)dS\right]\cos\left(\theta \right);\\ dS=2\pi R^{2}\sin\left(\theta \right)d\theta . \end{array}$$

The integration is done over the surface *S* of the moving sphere at time *t*, that is, for 0 ≤ *θ* ≤ *π*.

On the other hand, the expression of the *z* component Δ*X*_*σ*′*z*_^dr^ due to *σ*′(*R*, *θ*′, *t* − *t*_1_) is more subtle and depends on the movement of the charge. Indeed, if *vt*_1_ < *R*, *O*′ is inside the sphere of center *O* and of *R* radius; therefore, the image of the charge *σ*′ (which is a punctual charge concentrated in *O*′) is situated inside the cavity of center *O* and of radius *R*, and consequently:

(20) $$\begin{array}{c} \Delta {X_{\sigma^{\prime }z}}^{\text{dr}}=0,\;\text{for}\;\;{vt}_{1}<\;R. \end{array}$$

In contrast, for *vt*_1_ > *R*, *O*′ is outside the cavity, and therefore

(21) $$\begin{array}{c} \Delta {X_{\sigma^{\prime }z}}^{\text{dr}}=-Q\overset{\infty }{\underset{R/v}{\int }}\zeta \left(t_{1}\right){\left({vt}_{1}\right)}^{-2}dt_{1}. \end{array}$$

Now, the *z* component of the resulting dielectric frictional force *F*_*z*_^dr^ acting on the moving charged sphere due to the superficial charges *σ* and *σ*′ is therefore equal to

(22) $$\begin{array}{c} {F_{z}}^{\text{dr}}=Q\left(\Delta {X_{\sigma z}}^{\text{dr}}+\Delta {X_{\sigma^{\prime }z}}^{\text{dr}}\right)={F_{\sigma z}}^{\text{dr}}+{F_{\sigma^{\prime }z}}^{\text{dr}}. \end{array}$$

Calculation of *F*_*z*_^dr^ according to the different above equations leads to the following expressions which depend on the condition imposed on the velocity of the polyion.

(a) For *vt*_1_ < *R*,

(23) $$\begin{array}{c} {F_{\sigma^{\prime }z}}^{\text{dr}}=0. \end{array}$$

Therefore, *F*_*z*_^dr^ = *F*_*σz*_^dr^, and according to (19) and (22),

(24) Fzdr=(Q22R3)∫0R/vζ(t1)dt1 ×∫0πcos⁡(θ)sin(θ)[R+vt1cos⁡(θ)][1+2vt1cos⁡(θ)/R+(vt1/R)2]3/2  dθ.

The denominator *d* ^3^ = *R* ^3^[1 + 2*vt*_1_cos⁡(*θ*)/*R* + (*vt*_1_/*R*)^2^]^3/2^ must be always >0, so that when cos(*θ*) = −1, *d* = *R* − *vt*_1_, because *vt*_1_ < *R*. If we substitute the function cos(*θ*) by the new variable *x* = cos⁡(*θ*), we get

(25) Fzdr=(Q22R3)∫0R/vζ(t1)dt1×∫−1+1x[R+xvt1][1+2xvt1/R+(vt1/R)2]3/2  dx.

Now the integral

(26) $$\begin{array}{c} \overset{+1}{\underset{-1}{\int }}\frac{x\left[R+x{vt}_{1}\right]}{{\left[1+2x{vt}_{1}/R+{\left({vt}_{1}/R\right)}^{2}\right]}^{3/2}}dx \end{array}$$

can be decomposed as a sum of two integrals:

(27) R∫−1+1x[1+2xvt1/R+(vt1/R)2]3/2dx  +vt1∫−1+1x2[1+2xvt1/R+(vt1/R)2]3/2dx.

The integration over *x* involves elementary functions so that the above integrals exist in the literature. Since for *vt*_1_ < *R* and *x* = −1, we have *d* = *R* − *vt*_1_, the integration leads to the following exact results:

(28) R∫−1+1x[1+2xvt1/R+(vt1/R)2]3/2dx  =−2vt1[1−(vt1/R)2],vt1∫−1+1x2[1+2xvt1/R+(vt1/R)2]3/2dx  =2vt1[1+2(vt1/R)2]3[1−(vt1/R)2],

so that

(29) (Q22R3)∫−1+1x[R+xvt1][1+2xvt1/R+(vt1/R)2]3/2dx  =−(23)(Q2R3)vt1.

Consequently, (25) can be simplified as follows:

(30) $$\begin{array}{c} {F_{z}}^{\text{dr}}=-\left(\frac{2}{3}\right)\left(\frac{Q^{2}}{R^{3}}\right)\overset{R/v}{\underset{0}{\int }}\zeta \left(t_{1}\right){vt}_{1}dt_{1}. \end{array}$$

(b) For  *vt*_1_ > *R*.

In this case, when *x* = −1 we have *d* = *vt*_1_ − *R*, and integration leads to the following exact results:

(31) Fσzdr=(Q22R3)∫R/v∞ζ(t1)dt1×∫−1+1x[R+xvt1][1+2xvt1/R+(vt1/R)2]3/2dx,

with

(32) R∫−1+1x[1+2xvt1/R+(vt1/R)2]3/2dx  =2R{(vt1/R)2[1−(vt1/R)2]},vt1∫−1+1x2[1+2xvt1/R+(vt1/R)2]3/2dx  =−2R[2+(vt1/R)2]{3(vt1/R)2[1−(vt1/R)2]},

so that

(33) (Q22R3)∫−1+1x[R+xvt1][1+2xvt1/R+(vt1/R)2]3/2dx  =(Q23)(vt1)−2.

Consequently, (31) can be simplified as follows:

(34) $$\begin{array}{c} {F_{\sigma z}}^{\text{dr}}=\left(\frac{Q^{2}}{3}\right)\overset{\infty }{\underset{R/v}{\int }}\zeta \left(t_{1}\right){\left({vt}_{1}\right)}^{-2}dt_{1}. \end{array}$$

On the other hand, the use of (21), (22), and (34) leads to

(35) $$\begin{array}{c} {F_{\sigma^{\prime }z}}^{\text{dr}}=-Q^{2}\overset{\infty }{\underset{R/v}{\int }}\zeta \left(t_{1}\right){\left({vt}_{1}\right)}^{-2}dt_{1}. \end{array}$$

And therefore

(36) $$\begin{array}{c} {F_{z}}^{\text{dr}}=-\left(\frac{2Q^{2}}{3}\right)\overset{\infty }{\underset{R/v}{\int }}\zeta \left(t_{1}\right){\left({vt}_{1}\right)}^{-2}dt_{1}. \end{array}$$

Finally, the general expression of the resulting dielectric force: *F*_*z*_^dr^ which is valid for *vt*_1_ < *R* and for *vt*_1_ > *R* is

(37) Fzdr=−(23)(Q2R3)∫0R/vζ(t1)vt1dt1−(2Q23)∫R/v∞ζ(t1)(vt1)−2dt1.

This result is identical to original Zwanzig's result [7].

In the limit of low velocity that is *R*/*v* ~ *∞*, that is, for a slow particle

(38) Fzdr≈−(23)(Q2R3)∫0∞ζ(t1)vt1dt1=−(23)(vτ)(ε−ε∞)ε−2(Q)2(R)−3,

it is interesting to note that we can physically interpret this last result according to the linear response theory combined to a dimensional analysis as follows. Indeed, first, frictional effect is absent for immobile particle (*v* = 0); therefore, we expect that *F*_*z*_^dr^ must be proportional to *v*; second, there is no dielectric friction if the depolarization of the solvent molecules around the particle is instantaneous (*τ* = 0); consequently, *F*_*z*_^dr^ is also proportional to the relaxation time *τ*; on the other hand, there is no relaxation effect if the solvent is dielectrically saturated (*ε* = *ε*_*∞*_); consequently, *F*_*z*_^dr^ must be proportional to (*ε* − *ε*_*∞*_)*ε* ^−1^; finally, *F*_*z*_^dr^ is an electric force acting on a sphere of charge *Q* and radius *R*, and it is in principle proportional to (*Q*)^2^ *ε* ^−1^(*R*)^−2^; now as *F*_*z*_^dr^ is also proportional to the length *vτ*, it follows that *F*_*z*_^dr^ must be proportional to (*R*)^−3^ so that it sharply decreases with the size of the particle.

More rigorous derivations of *F*_*z*_^dr^ taking into account hydrodynamic motion of the solvent were performed successively by Zwanzig [7], Hubbard and Douglas [17], and Hubbard and Onsager [18]. More recent approach taking into account molecular correlation between solvent molecules and moving particle was presented by Wolynes [19]. However, if the charged sphere is assumed to be a conductor of large radius *R*, then hydrodynamic effects become small and all theories converge to Zwanzig's original result.

Note finally that (38) has been generalized to the case of ellipsoidal polyion of *eZ*_*S*_ charge characterized by an apparent radius *R*_app_ which depends on the eccentricity *γ* so that *R*_app_ → *R* when *γ* → 0 [6]; the result is

(39) $$\begin{array}{c} {F_{z}}^{\text{dr}}=-\left(\frac{2}{3}\right)\left(v\tau \right)\left(\varepsilon -\varepsilon_{\infty }\right)\varepsilon^{-2}{\left(eZ_{S}\right)}^{2}{\left(R_{\text{app}}\right)}^{-3}. \end{array}$$

## 3. General Expression of the Dielectric Frictional Force

The previous expression giving the dielectric frictional force *F*_*z*_^dr^ acting on an ellipsoidal (or spherical) polyion of center *O*, *A* and *B* focuses, *R* minor axis, *a*_*R*_ major axis, *L* length (*L* = *AB*), and *eZ*_*S*_ charge, can be found by applying the following general method [6, 14].

**Figure 3:**
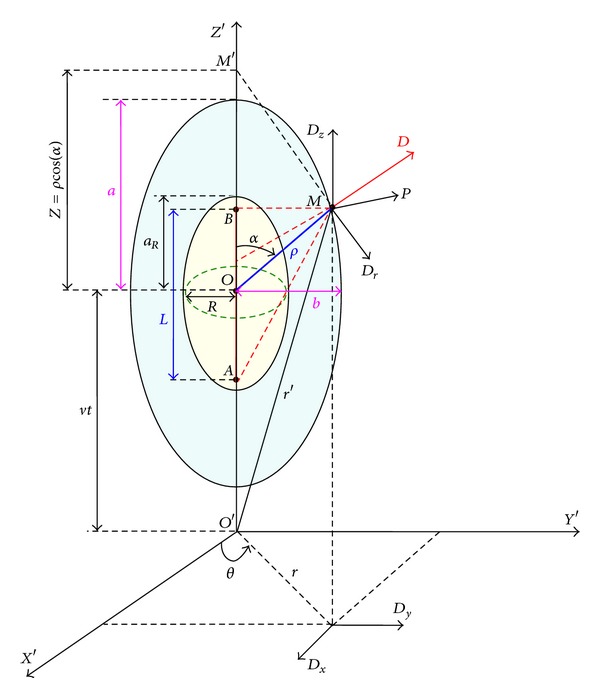
Representation of a moving ellipsoidal polyion, surrounded by its dielectric medium.

As explained above, the *time dependent* polarization **P**(**r**′, *t*) at a position **r**′ around the polyion (*M* point; see Figure 3) is induced by the displacement field **D**(**r**′, *t* − *t*_1_) due to the (*eZ*_*S*_) charge of the moving polyion. In its turn, this induced polarization creates a reacting dielectric relaxation field Δ**Χ** ^dr^ in *O* and therefore exerts a dielectric frictional force (**F** ^dr^ = *eZ*_*S*_Δ**Χ** ^dr^) back on the polyion directed along the *OZ* axis. Indeed, according to the dielectric theory, the local charge density of the dielectric molecules into the element of volume *dx* *dy* *dz* at *M* is equal to −∇·**P**(**r**′, *t*) so that

(40) $$\begin{array}{c} \Delta X^{\text{dr}}={∭}_{}^{}\frac{\nabla \cdot P\left(r^{\prime },t\right)\rho }{\rho^{3}}dx\;dy\;dz;\;\text{with}:\left(\vec{\rho }=OM\right), \end{array}$$

with

(41) $$\begin{array}{c} P\left(r^{\prime },t\right)=\left(\frac{1}{4\pi }\right)\overset{\infty }{\underset{0}{\int }}\zeta \left(t_{1}\right)D\left(r^{\prime },t-t_{1}\right)dt_{1}. \end{array}$$

The explicit development of the previous relations according to adequate boundary conditions enables us to reduce the expression of the dielectric frictional force **F** ^dr^ to the following general form which is in fact valid for a charged macroion of any shape:

(42) Fzdr=(eZS)ΔXzdr=(eZS4π)∫0∞  ζ(t1)IOz(t1)dt1.

*I* ^*O*^_*z*_(*t*_1_) is the so-called key integral [6] related to the components *D*_*x*_, *D*_*y*_, and *D*_*z*_ of **D**(**r**′, *t* − *t*_1_) as follows:

(43) IOz(t1) =∭[−Dz(t−t1)ρ3+3Z2Dz(t−t1)ρ5     +3xZDx(t−t1)ρ5+3yZDy(t−t1)ρ5]dx dy dz.

According to Figure 3, *x*, *y* and *z* are the Cartesian coordinates of the vector radius **r**′ of module *O*′*M*; *ρ* is the vector radius of module *ρ* = *OM*. *O*′*O* = *vt* is the distance covered by the center *O* of the moving polyion during the time *t* with the velocity *v*, and *Z* = *z* − *vt* = *ρ*cos⁡(*α*).

The above triple integration is effectuated over the whole volume of the dielectric medium; consequently, the main mathematical difficulty comes from the limiting conditions imposed on the interdependent lower boundaries of integration (*x*°, *y*°, *z*°) defining the *finite* surface region surrounding the volume from which the dielectric medium must be excluded.

Demonstration of (42) and (43) was first achieved by Zwanzig in its original work [7] in the case of a moving spherical charge and then by the authors of [6] in the case of a moving ellipsoidal polyion.

As indication, we give below the explicit formulas corresponding to the components *D*_*x*_, *D*_*y*_, and *D*_*z*_, created by an ellipsoidal polyion:

(44) Dx(t−t1) =−λr−1cos⁡θ  ×{[Z+(vt1−L/2)][ρ2+2Z(vt1−L/2)+(vt1−L/2)2]1/2   −[Z+(vt1+L/2)][ρ2+2Z(vt1+L/2)+(vt1+L/2)2]1/2},Dy(t−t1) =−λr−1sinθ  ×{[Z+(vt1−L/2)][ρ2+2Z(vt1−L/2)+(vt1−L/2)2]1/2   −[Z+(vt1+L/2)][ρ2+2Z(vt1+L/2)+(vt1+L/2)2]1/2},Dz(t−t1) =λ{1[ρ2+2Z(vt1−L/2)+(vt1−L/2)2]1/2   −1[ρ2+2Z(vt1+L/2)+(vt1+L/2)2]1/2}.

The parameters *vt*, *L*, *r*, *Z*, *ρ*, *α*, and *θ* are defined in Figure 3; and *λ* = (*eZ*_*S*_)*L* ^−1^ is the linear charge density of the polyion. The introduction of the explicit expressions of *D*_*x*_, *D*_*y*_, and *D*_*z*_, into (43) leads to the following expression of the key integral:

(45) $$\begin{array}{c} {I^{O}}_{z}\left(t_{1}\right)=-\left(\frac{8\pi }{3}\right)\left(\frac{eZ_{S}}{{R_{\text{app}}}^{3}}\right){vt}_{1}. \end{array}$$

Therefore, integration of *I* ^*O*^_*z*_(*t*_1_) according to (42) leads to the same results obtained in paragraph 2 ((38) and (39)).

In the next paragraph, we will generalize this approach based on the notion of the key integral *I*, in order to calculate in details the dielectric frictional force acting on a moving chain of charged spheres.

## 4. Expression of the Dielectric Frictional Force on a Moving Chain of Charged Spheres

**Figure 4:**
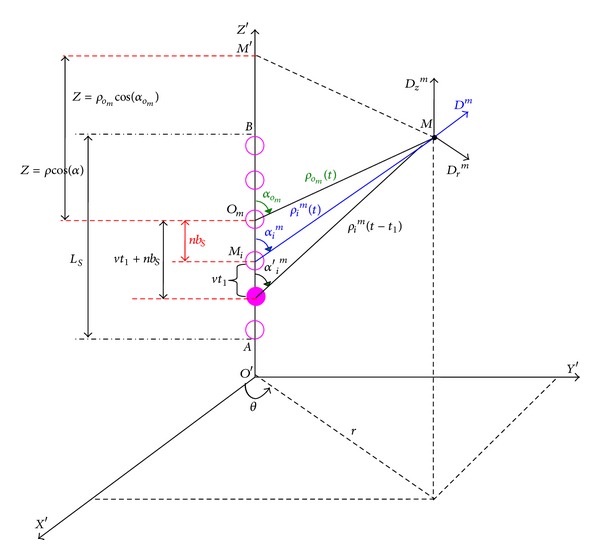
Representation of a moving polyion as a chain of successive charged spheres.

Figure 4 represents a moving polyion as a succession of |*Z*_*S*_| identical charged rigid spheres (or “groups”) of centers *M*_1_, *M*_2_,…, *M*_*i*_,…, *M*_|*Z*_*S*_|_ of *R*_*g*_ radius and *q*_*i*_ charge. The distance of separation between two successive charged spheres is defined by *b*_*S*_ = *L*_*S*_/|*Z*_*S*_ | ≥2*R*_*g*_.

*O*′*X*′*Y*′*Z*′ indicates the fixed Cartesian reference frame so that the chain moves along *OZ*′ with a velocity *v*. The relative position Δ*z*_*i*_ of a charged sphere “*i*” of center *M*_*i*_ is defined compared to an *arbitrary* origin *O*_*m*_ which coincides with the center of an unspecified sphere “*m*” so that

(46) $$\begin{array}{c} \Delta z_{i}\equiv z_{O_{m}}-z_{M_{i}}. \end{array}$$

*z*_*M*_*i*__ and *z*_*O*_*m*__ are, respectively, the *z* coordinates of *M*_*i*_ and *O*_*m*_. Now, if Δ*z*_*i*_>0, we can write

(47) $$\begin{array}{c} \Delta z_{i}\equiv \Delta z_{n}=nb_{S},\;\text{with}\;\;n=1,2,3,\ldots ,N. \end{array}$$

If Δ*z*_*i*_ < 0, then

(48) $$\begin{array}{c} \Delta z_{i}\equiv \Delta z_{n^{\prime }}=-n^{\prime }b_{S},\;\text{with}\;\;n^{\prime }=1,2,3,\ldots ,N^{\prime }, \end{array}$$

with the obvious condition

(49) $$\begin{array}{c} N+N^{\prime }=\left|Z_{S}\right|-1. \end{array}$$

*M*(*x*′, *y*′, *z*′) is an arbitrary point inside the dielectric medium and *ρ*_*O*_*m*__(*t*) and *ρ*_*i*_^*m*^(*t*) are the distances of separation between *M* and, respectively, *O*_*m*_ and *M*_*i*_ at time *t*. On the other hand, *ρ*_*i*_^*m*^(*t* − *t*_1_) is the distance of separation between *M* and *M*_*i*_ at time (*t* − *t*_1_) (i.e., the center of the red sphere represented in Figure 4), so that *vt*_1_ is equal to the distance of separation between the center of the sphere “*i*” at time *t* and its center at (*t* − *t*_1_).

According to Figure 4, in which Δ*z*_*i*_ > 0, the distance *ρ*_*i*_^*m*^(*t* − *t*_1_) is equal to

(50) [ρim(t−t1)]2=ρOm2+(vt1+nbs)2+2ρOm(vt1+nbs)cos⁡⁡(αOm)≡[au+b].

For simplification we have used the new parameters

(51) $$\begin{array}{c} r=\rho_{O_{m}}\sin\left(\alpha_{O_{m}}\right);\;\;u=\cos\left(\alpha_{O_{m}}\right);\\ a=2\rho_{O_{m}}\left({vt}_{1}+nb_{S}\right);\;\;b={\rho_{O_{m}}}^{2}+{\left({vt}_{1}+nb_{S}\right)}^{2}. \end{array}$$

Now, the sphere “*i*” of charge *q*_*i*_ creates in the point *M* at (*t* − *t*_1_) an electrical displacement field

(52) $$\begin{array}{c} {D_{i}}^{m}\left(t-t_{1}\right)=q_{i}\left\{\frac{{\rho_{i}}^{m}\left(t-t_{1}\right)}{{\rho_{i}}^{m}\left(t-t_{1}\right)}\right\}{\left[{\rho_{i}}^{m}\left(t-t_{1}\right)\right]}^{-2}. \end{array}$$

Therefore, the expressions of the components *D*_*xi*_^*m*^(*t* − *t*_1_), *D*_*yi*_^*m*^(*t* − *t*_1_) and *D*_*zi*_^*m*^(*t* − *t*_1_) are

(53) $$\begin{array}{c} {D_{xi}}^{m}\left(t-t_{1}\right)=\frac{q_{i}r\cos\left(\theta \right)}{{\left[{\rho_{i}}^{m}\left(t-t_{1}\right)\right]}^{3}}=q_{i}r\cos\left(\theta \right){\left[au+b\right]}^{-3/2}, \end{array}$$

(54) $$\begin{array}{c} {D_{yi}}^{m}\left(t-t_{1}\right)=\frac{q_{i}r\sin\left(\theta \right)}{{\left[{\rho_{i}}^{m}\left(t-t_{1}\right)\right]}^{3}}=q_{i}r\sin\left(\theta \right){\left[au+b\right]}^{-3/2}, \end{array}$$

(55) Dzim(t−t1)=qiρim(t−t1)cos⁡(α′im)[ρim(t−t1)]3=qim[ρOmu+vt1−nbs][au+b]−3/2.

It is also important to note that the denominator [*au* + *b*] which appears in (53)–(55) must be strictly positive.

According to the principle of superposition, the total displacement field **D**(*x*′, *y*′, *z*′, *t* − *t*_1_)due to total charge of the moving polyion in *M* at (*t* − *t*_1_) is equal to the sum of the local fields **D**_*i*_^*m*^(*t* − *t*_1_) created by the successive spherical charges *q*_*i*_:

(56) $$\begin{array}{c} D\left(x^{\prime },y^{\prime },z^{\prime },t-t_{1}\right)=\overset{}{\underset{i}{\sum }}{D_{i}}^{m}\left(t-t_{1}\right). \end{array}$$

It is clear that the components of **D**_*O*_*m*__(*t* − *t*_1_) created by the reference charge *O*_*m*_ in *M* at *t* − *t*_1_ are calculated from (52)–(55) by imposing Δ*z*_*i*_ = 0. We can also develop the equation of superposition given by (52) as follows:

(57) D(x′,y′,z′,t−t1)=DOm(t−t1)+∑n=1NDnm(t−t1)+∑n′=1N′Dn′m(t−t1).

Now, in order to simplify calculations of the dielectric frictional force according to the general method exposed in Section 3 on the basis of (42) and (43), we will define for each number *n* (or *n*′) a corresponding *cross* key integral *I*_*z*_^*O*_*m*_^_*n*_(*t*_1_) as follows:

(58) IzOmn(t1) =∭{−Dznm(t−t1)ρOm3+3Z2Dznm(t−t1)ρOm5     +3x′ZDxnm(t−t1)ρOm5     +3y′ZDynm(t−t1)ρOm5}dx′dy′dz′,

with *Z* = *ρ*_*O*_*m*__ *u*. The triple integration in this equation is done over the whole volume except the volume of the spherical charge *O*_*m*_. The explicit expression of this integral in terms of the spherical coordinates *ρ*_*O*_*m*__ and *u* = cos⁡(*α*_*O*_*m*__) is obtained after replacing the volume element of the dielectric  *dx*′*dy*′*dz*′ by −*ρ*_*O*_*m*__^2^ *du* *dρ*_*O*_*m*__ *dθ*. The result is

(59) IzOmn(t1)=2π∫Rg∞ρOm−1dρOm∫−1+1fn°(ρOm,u)du,fn°(ρOm,u) ={−Dmzn(t−t1)+3Z2Dmzn(t−t1)ρOm2   +3x′ZDmxn(t−t1)ρOm2+3y′ZDmyn(t−t1)ρOm2}.

The insertion of the expressions of *D*_*xn*_^*m*^(*t* − *t*_1_), *D*_*yn*_^*m*^(*t* − *t*_1_), and *D*_*zn*_^*m*^(*t* − *t*_1_) given by (53)–(55) into the above relations leads to

(60) IzOmn(t1) =2πqn∫Rg∞ρOm−1dρOm  ×∫−1+1[au+b]−3/2{2ρOmu+(vt1+nbS)(3u2−1)}du.

We can decompose the second integral over the variable *u* as a sum of three elementary integrals:

(61) ∫−1+1[au+b]−3/2{2ρOmu+(vt1+nbS)(3u2−1)}du  =[Io1(ρOm)+Io2(ρOm)+Io3(ρOm)],Io1(ρOm)=−(vt1+nbs)∫−1+1[au+b]−3/2du,Io2(ρOm)=2ρOm∫−1+1[au+b]−3/2udu,Io3(ρOm)=3(vt1+nbs)∫−1+1[au+b]−3/2u2du.

At this stage, we must distinguish two conditions.

(a) *ρ*_*O*_*m*__ ≥ *vt*_1_ ± *nb*_*S*_, so that (*a*+*b*)^1/2^ = *ρ*_*O*_*m*__ + (*vt*_1_ + *nb*_*S*_) and (*b*−*a*)^1/2^ = *ρ*_*O*_*m*__ − (*vt*_1_ + *nb*_*S*_); thus,

(62) Io1(ρOm)=−2ρOm−1(vt1+nbS)[ρOm2−(vt1+nbS)2]−1,Io2(ρOm)=−4ρOm−1[ρOm2−(vt1+nbS)2]−1(vt1+nbS),Io3(ρOm)=2ρOm−3(vt1+nbS)[ρOm2−(vt1+nbS)2]−1×[ρOm2+2(vt1+nbS)2].

Therefore

(63) ∫−1+1[au+b]−3/2{2ρOmu+(vt1+nbS)(3u2−1)}du  =−4ρOm−3(vt1+nbS).

(b) *ρ*_*O*_*m*__ ≤ *vt*_1_ ± *nb*_*S*_, so that (*a*+*b*)^1/2  ^ = *ρ*_*O*_*m*__ + (*vt*_1_ + *nb*_*S*_) and (*b*−*a*)^1/2^ = −*ρ*_*O*_*m*__ + (*vt*_1_ + *nb*_*S*_); thus,

(64) Io1(ρOm)=2[ρOm2−(vt1+nbS)2]−1,Io2(ρOm)=4ρOm2(vt1+nbS)−2[ρOm2−(vt1+nbS)2]−1,Io3(ρOm)=−2{2ρOm2+(vt1+nbS)2}×(vt1+nbS)−2[ρOm2−(vt1+nbS)2]−1.

Therefore

(65) $$\begin{array}{c} \overset{+1}{\underset{-1}{\int }}{\left[au+b\right]}^{-3/2}\left\{2\rho_{O_{m}}u+\left({vt}_{1}+nb_{S}\right)\left(3u^{2}-1\right)\right\}du=0. \end{array}$$

This last equation means that polarized solvent molecules inside the sphere of radius *ρ*_*O*_*m*__ = (*vt*_1_ + *nb*_*S*_) do not participate in the dielectric friction. (Note that we have obtained a similar result in the case of an ellipsoidal polyion of center *O* and length *L* [6]. All occur as if during its translational motion, the polyion turns around its center so that the solvent molecules inside the sphere of center *O* and *L*/2 radius do not take part in the process of dielectric friction).

Finally, by introducing (63) and (65) into (60) and after simple integration we obtain the following final expression of the cross key integral *I*_*z*_^*O*_*m*_^_*n*_(*t*_1_):

(66) IzOmn(t1)=−8πqn(vt1+nbS)∫vt1+nbs∞dρOmρOm4=−(8π3)qn(vt1+nbS)−2.

It is important to underline that *I*_*z*_^*O*_*m*_^_*n*_(*t*_1_) represents a *cross *(*n*, *m*) effect. Indeed, it is proportional to the dielectric frictional force (*F*_*z*_^dr^)^*O*_*m*_^_*n*_ acting on the reference charge *O*_*m*_, due to the polarizationof the solvent by the field **D**_*n*_^*m*^(*t* − *t*_1_) created by the spherical charge “*q*_*n*_”:

(67) $$\begin{array}{c} {{\left({F_{z}}^{\text{dr}}\right)}^{O_{m}}}_{n}=\left(\frac{q_{O_{m}}}{4\pi }\right)\overset{\infty }{\underset{0}{\int }}\zeta \left(t_{1}\right){{I_{z}}^{O_{m}}}_{n}\left(t_{1}\right)dt_{1}. \end{array}$$

In the case of a spherical charge “*q*_*n*′_” characterized by the condition Δ*z*_*n*′_ = −*n*′*b*_*S*_ < 0, we define in the same way a key integral *I*_*z*_^*O*_*m*_^_*n*′_(*t*_1_) related to its corresponding force (*F*_*z*_^dr^)^*O*_*m*_^_*n*′_:

(68) IzOmn′(t1)=−8πqn′(vt1−n′bS)∫n′bs−vt1∞dρOmρOm4=(8π3)qn′(vt1−n′bS)−2

with the condition *n*′*b*_*S*_>*vt*_1_.

In contrast, the *self*-key integral *I*_*z*_^*O*_*m*_^_*m*_(*t*_1_) is proportional to the *self*-dielectric frictional force (*F*_*z*_^dr^)^*O*_*m*_^_*m*_ acting on the reference charge *O*_*m*_ which is induced by its *own* charge *q*_*O*_*m*__. *I*_*z*_^*O*_*m*_^_*m*_(*t*_1_) is therefore Zwanzig's integral relative to a moving sphere of *q*_*O*_*m*__ charge and *R*_*g*_ radius; its expression is therefore analogous to (45):

(69) $$\begin{array}{c} {{I_{z}}^{O_{m}}}_{m}\left(t_{1}\right)=-\left(\frac{8\pi }{3}\right)\left(\frac{q_{O_{m}}}{{R_{g}}^{3}}\right){vt}_{1}. \end{array}$$

Consequently, the total dielectric force (*F*_*z*_^dr^)^*O*_*m*_^ acting on the reference spherical charge *O*_*m*_ which is induced by the different spherical charges of the chain is given by

(70) (Fzdr)Om =(qOm4π)∫0∞ζ(t1)[IzOmm(t1)+∑n=1NIzOmn(t1)          +∑n′=1N′IzOmn′(t1)]dt1.

The resulting dielectric force *F*_*z*_^dr^ acting on the polyion is obtained via the total key integral *I*_*z*_(*t*_1_) by a summation over the |*Z*_*S*_| reference spherical charges *O*_*m*_:

(71) $$\begin{array}{c} \left({F_{z}}^{\text{dr}}\right)\equiv \left(\frac{q_{O_{m}}}{4\pi }\right)\overset{\infty }{\underset{0}{\int }}\zeta \left(t_{1}\right)I_{z}\left(t_{1}\right)dt_{1}=\overset{\left|Z_{S}\right|}{\underset{m=1}{\sum }}{\left({F_{z}}^{\text{dr}}\right)}^{O_{m}}, \end{array}$$

(72) Iz(t1)=∑m=1|ZS|[IzOmm(t1)+∑n=1NIzOmn(t1)       +∑n′=1N′IzOmn′(t1)].

Recall that *N* and *N*′ are related by the condition +*N*′ = |*Z*_*S*_ | −1. According to (66), (68), and (69), the key integrals *I*_*z*_^*O*_*m*_^_*m*_(*t*_1_), *I*_*z*_^*O*_*m*_^_*n*_(*t*_1_), and *I*_*z*_^*O*_*m*_^_*n*′_(*t*_1_) are, respectively, proportional to −*vt*_1_/*R*_*g*_^3^, −(*vt*_1_+*nb*_*S*_)^−2^, and (*vt*_1_−*n*′*b*_*S*_)^−2^; one can therefore regroup the different terms of the above sums over *n* and *n*′ in order to transform them into a sum of couples (*n* = *p*, *n*′ = *p*) of the form [(*vt*_1_+*pb*_*S*_)^−2^ − (*vt*_1_−*pb*_*S*_)^−2^]; each couple has a “degeneracy” equal to (|*Z*_*S*_ | −*p*). Consider

(73) Iz(t1) =−(8πqOm3)  ×{|ZS|vt1Rg3+∑p=1(|ZS|−1)(|ZS|−p)         ×[(vt1+pbS)−2−(vt1−pbS)−2]}.

Table 1 gives as an example the matrix of the various terms of the key integral *I*_*z*_(*t*_1_) of a chain of eight (|*Z*_*S*_ | = 8) charged spheres.

In the approximation of the linear response, *I*_*z*_(*t*_1_) must be proportional to (*vt*_1_) with the condition *pb*_*S*_ > *vt*_1_; its expression can therefore be simplified as follows:

(74) Iz(t1) =−(8πqOm3)vt1{|ZS|Rg3−4bS−3∑p=1(|ZS|−1)p−3(|ZS|−p)}.

As the sums ∑*p* ^−2^ and ∑*p* ^−3^ converge rapidly, respectively, to

(75) $$\begin{array}{c} \overset{\infty }{\underset{p=1}{\sum }}p^{-2}=\frac{\pi^{2}}{6}\approx 1.645;\;\;\overset{\infty }{\underset{p=1}{\sum }}p^{-3}\approx 1.202, \end{array}$$

the final result for large |*Z*_*S*_| is therefore

(76) Iz(t1)≈−(8πqOm3)vt1×{|ZS|Rg−3−4bS−3[1.202|ZS|−1.645]}.

We now define the “*interference factor*” *f* as follows:

(77) f=(bSRg)≥2,Iz(t1)≈−(8πqOm3)vt1bS−3×{|ZS|f3−4[1.202|ZS|−1.645]}.

The final expression of the dielectric force *F*_*z*_^dr^ acting on the charged chain of the polyion is obtained according to (76), (71), and (16) and by using the relation

(78) ∫ζ(t1)vt1dt1=(vτ)(ε−ε∞)ε−2,(Fzdr)=(qOm4π)∫0∞ζ(t1)Iz(t1)dt1=−(23)(qOm)2(vτ)bS−3(ε−ε∞)ε−2 ×{|ZS|(f3−4.808)+6.58}.

Note that when the distance of separation *b*_*S*_ between two successive charges is large compared to the radius *R*_*g*_ of the charged groups (i.e., *b*_*S*_ ≫ *R*_*g*_ and *f* ≫ 2), the mutual influence between charged spheres vanishes, so that the solvent molecules surrounding each sphere “*i*” are polarized essentially by the displacement field **D**_*i*_^*m*^(*t* − *t*_1_) caused by its own charge *q*_*i*_ (*no interference*). Consequently, the dielectric force:  (*F*_*z*_^dr^)_*i*_ undergone by each group “*i*” is therefore reduced to Zwanzig's force −(2/3)(*q*_*O*_*m*__)^2^(*vτ*)*R*_*g*_^−3^(*ε* − *ε*_*∞*_)*ε* ^−2^, and the total force *F*_*z*_^dr^ acting on the polyion is obtained by superposition of these |*Z*_*S*_| Zwanzig's forces:

(79) (Fzdr)f→∞=(qOm4π)∫ζ(t1)Iz(t1)dt1=−|ZS|(23)(qOm)2(vτ)−3Rg−3(ε−ε∞)ε−2.

It is also important to realize that in all cases *F*_*z*_^dr^ ~ −*v*, which means that the total force *F*_*z*_^dr^ is always opposed by the movement of the polyion; however, this braking effect is attenuated by the effect of interference, as it is shown by the ratio

(80) (Fzdr)(Fzdr)f→∞={(1−4.808f−3)+6.58|ZS|−1f−3}<1;f≥2.

It is now possible to derive the expression of the dielectric friction coefficient defined previously by *β* ^df^_*P*_ = |Δ**Χ** ^dr^ | /|**E**|. Δ**Χ** ^dr^ is the local field caused by the polarization of the solvent molecules around the moving polyion and it is related to the frictional force **F** ^dr^ as follows:

(81) $$\begin{array}{c} F^{\text{dr}}=\left(eZ_{\text{app}}\right)\Delta X^{\text{dr}}. \end{array}$$

(*eZ*_app_) is the effective (apparent) electric charge of the polyion equal to

(82) $$\begin{array}{c} \left(eZ_{\text{app}}\right)=q\left|Z_{S}\right|. \end{array}$$

*q* ≡ *q*_*O*_*m*__ is the effective charge of any charged sphere *O*_*m*_ of the chain. On the other hand, the velocity *v* of the polyion is related to its electrical mobility *u*_*P*_ = (*λ*_*P*_/*F*) and to the external field **E** (directed along the *z*-axis), according to

(83) $$\begin{array}{c} v=u_{P}\text{E}. \end{array}$$

Introduction of (83) into (78)–(82) leads to the general expression of the dielectric friction coefficient *β* ^df^_*P*_:

(84) βdfP=(23)|q|(uPτ)bS−3(ε−ε∞)ε−2×{(f3−4.808)+|ZS|−16.58}.

## 5. Coupling between the Dielectric Friction Effect and the Ionic Condensation Processes

Because of the ionic condensation effect, the charged groups of the polyion are partially neutralized by the counterions “*k*” of *eZ*_*k*_ charge and *R*_*k*_ effective radius. The degree of condensation (1 − *α*) depends on the configuration of the polyion. Recall that according to Manning's rod-like limiting model (*R*_*C*_/*L*_*S*_ → 0) [5], the degree of ionic condensation (1 − *α*_*M*_) is independent of the counterions concentration *C*_*k*_ so that *α*_*M*_ = *b*_*S*_/|*Z*_*k*_ | *L*_*B*_; where *L*_*B*_ = *e* ^2^/*εk*_*B*_ *T* is the Bjerrum length, *k*_*B*_ is the Boltzmann constant. In fact, experimental conductivity results are not in general in conformity with Manning's theory [1–5]. In particular, we have proved that for more realistic polyelectrolyte models the degree of dissociation *α* obeys in general Ostwald's principle of dilution and consequently *α* → 1 when *C*_*k*_ → 0. The degree *α* was calculated on the basis of the two-state model [6] (double layer) proposed by Dobrynin and Rubinstein [20] (see Figure 1) and by the generalization of the theory of ionic association of Fuoss [8, 9], so that *α* = *α*_Fuoss_ for spherical polyion (*L* → 0), and *α* ≈ *α*_*M*_ for an infinite chain (*L* → *∞*). The result of an ellipsoidal polyion (*L* < *L*_*S*_) of concentration *C*_*P*_ and of volume *V*_*P*_ is [6, 12]

(85) $$\begin{array}{c} \frac{\left(1-\alpha \right)}{\alpha }={\left(1-V_{P}C_{P}\right)}^{-1}V_{P}C_{P}{\left[\frac{g\left(R,L\right)}{g\left(d,L\right)}\right]}^{\left\{(\alpha +|Z_{i}/Z_{S}|)/\alpha_{M}\right\}}. \end{array}$$

The degree (1 − *α*) of ionic condensation on a charged chain is obtained from this relation by replacing *L* by the structural length *L*_*S*_ and *R* by *R*_*g*_. On the other hand, as (*eZ*_app_) = *eαZ*_*S*_, the charge *q* is thus equal to

(86) $$\begin{array}{c} q=e\alpha \left(\frac{\left|Z_{S}\right|}{Z_{S}}\right). \end{array}$$

As indicated in Section 1, the electrical mobility *u*_*P*_ = (*λ*_*P*_/*F*) of the polyion is a complex function of *α*, and its expression can be written in the following general form:

(87) $$\begin{array}{c} u_{P}\approx \alpha {u_{P}}^{{}^{\circ}\text{HD}}{\left(1+\beta^{\text{ir}}\right)}^{-1}\frac{\left[1-\left(\langle R\rangle /\langle d\rangle \;\right)\right]}{\left(1+{\beta_{P}}^{\text{df}}\right)};\\ {u_{P}}^{{}^{\circ}\text{HD}}=\left(\frac{e\left|Z_{S}\right|}{6\pi \eta \langle R\rangle }\right). \end{array}$$

The insertion of these last equations into the expression of the coefficient of dielectric friction *β* ^df^_*P*_ leads after linearization to

(88) βdfP=(2α2τ3)(e2|ZS|6πη〈R〉)bS−3(ε−ε∞)ε−2×{(f3−4.808)+|ZS|−16.58}×[1−(〈R〉〈d〉)].

Now, if we replace the relaxation time *τ* by its Debye's expression: *τ* = (6*πηR*_*w*_^3^/*k*_*B*_ *T*), with *L*_*B*_ = *e* ^2^/*εk*_*B*_ *T*, the general explicit expression of *β* ^df^_*P*_ becomes

(89) βdfP=(2α23)|ZS|(Rwbs)3(LB〈R〉)×[1−(〈R〉〈d〉)][1−ε∞ε]×{(f3−4.808)+|ZS|−16.58}.

This equation shows that the coefficient of dielectric friction *β* ^df^_*P*_ corresponding to a chain of charged sphere is as expected and in a first approximation proportional to number |*Z*_*S*_| of charged groups and to the square power of the degree *α* of dissociation *β* ^df^_*P*_ = *α* ^2^ *β*°_*P*_^df^.

Comparison between the (*β* ^df^_*P*_)_chain_ given by (89) and (*β* ^′df^_*P*_)_ellipsoid_ given by (10) leads to

(90) (βdfP)chain(β′dfP)ellipsoide~(αα′)2|ZS|−1(R′appbs)3(〈R′〉〈R〉)×{(f3−4.808)+|ZS|−16.58}.

This ratio explains the important increase of the dielectric friction during the transition from an ellipsoid configuration toward the stretched chain configuration. Indeed, as  *f* ^3^ ~ 8 and *R*_app_′ ~ (*L*_*S*_/2), |*Z*_*S*_|^−1^(*R*_app_′/*b*_*s*_)^3^ ~ (|*Z*_*S*_|^2^/8), and therefore

(91) $$\begin{array}{c} \frac{{\left({\beta^{\text{df}}}_{P}\right)}_{\text{chain}}}{{\left({\beta^{\prime \text{df}}}_{P}\right)}_{\text{ellipsoide}}}\sim \left(\frac{{\left|Z_{S}\right|}^{2}}{2}\right)\gg 1. \end{array}$$

Note finally that according to (89), *β* ^df^_*P*_ increases with dilution (*d* → *∞*) in the case of polyions obeying Oswald's regime (*α* → 1) and it remains sensibly constant for polyions obeying Manning's regime for which *α* is constant.

## 6. Variation of the Conductibility of a Polyion with Dilution

As indicated in the previous sections, the dilution increases the degree *α* of ionic dissociation and the mean radius 〈*d*〉 of the ionic atmosphere and modifies the configuration of the polyion. However, for polyions obeying Manning's regime, the degree “*α*” remains sensibly constant, so that both frictional coefficients *β*_*P*_^df^ and *β* ^ir^ are also constants. In this case, it is therefore easier to test the importance of the dielectric friction effect on stretched polyions by comparing the conductibility Λ_poly_ of such polyelectrolytes to the one calculated in absence of dielectric friction. Recall that according to limiting Manning's model the dielectric friction is nonexistent, *β*_*P*_^df^ = 0; indeed, the moving polyion is assumed to be an infinite thread with a continuous distribution of charge, so that it seems immobile and therefore the polarization of its surrounding solvent molecules is not perturbed by the movement.

**Figure 5:**
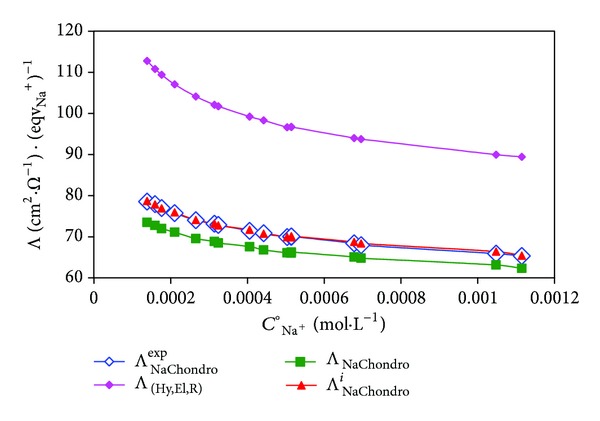
Comparison between variations with *C*_Na^+^_ of experimental (Λ^exp⁡^_NaChondro_, blue 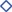 points) and theoretical equivalent conductivities (Λ_Hy,El,R_ without dielectric friction; Λ_NaChondro_ with dielectric friction in absence of interference; Λ^*i*^_NaChondro_ with dielectric friction in presence of interference) of sodium chondroitin sulfate in water at 25°C.

As an illustration, Figure 5 compares the variation (with the total concentration *C*°_Na^+^_) of the experimental conductibility  Λ^exp⁡^_NaChondro_ and (blue 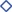 points) of sodium chondroitin sulfate in water at 25°C to the following three theoretical conductibility types: Λ_Hy,El,R_, Λ_NaChondro_ and Λ^*i*^_NaChondro_. The first one is calculated in absence of dielectric friction. The second is calculated by taking into account the dielectric friction but in absence of interference. The last one is calculated with dielectric friction in presence of interference.

The structural characteristics of this polyion are [6, 7] as follows. *Z*_*S*_ = −75 ± 3 is the structural charge number. *L*_*S*_ = 435 ± 15 Å is the structural length. *b*_*S*_ = 5.8 ± 0.2 Å is the charge-to-charge distance. *R*_*C*_ = 6 ± 0.5 Å is the cylindrical radius of the polyion chain.

Sodium chondroitin sulfate is one of peculiar polyelectrolytes for which the behavior of ionic condensation in aqueous solution is compatible to the model of Manning [13, 14], so that *α* remains practically constant equal to *α* = *α*_*M*_ = *b*_*S*_/*L*_*B*_ = 0.81.

The equivalent conductivity Λ_Poly_ of this polyelectrolyte is therefore equal to

(92) $$\begin{array}{c} \Lambda_{\text{Poly}}=\alpha_{M}\left(\lambda_{P}+\lambda_{{\text{Na}}^{+}}\right). \end{array}$$

*λ*_*P*_ and *λ*_Na^+^_ depend on the concentration of the free counterions *α*_*M*_ *C*°_Na^+^_ because of the braking effects due to the ionic atmosphere, giving rise to the electrophoretic effect (Δ*λ* ^el^) and to the ionic relaxation effect (*β* ^ir^). The explicit expressions of *λ*_Na^+^_ and *λ*_*P*_ in terms of *α* = *α*_*M*_, Δ*λ* ^el^, *β* ^ir^, and *β*°_*P*_^df^ are the following:

(93) $$\begin{array}{c} \lambda_{{\text{Na}}^{+}}=\frac{\left({\lambda^{{}^{\circ}}}_{{\text{Na}}^{+}}-\Delta {\lambda_{{\text{Na}}^{+}}}^{\text{el}}\right)}{\left(1+{\beta_{{\text{Na}}^{+}}}^{\text{ir}}\right)};\\ \Delta {\lambda_{{\text{Na}}^{+}}}^{\text{el}}=\left(\frac{Fe}{6\pi \eta d_{{\text{Na}}^{+}}}\right);\;\;d_{{\text{Na}}^{+}}=\frac{R_{{\text{Na}}^{+}}+1}{2\Gamma_{\text{MSA}}}. \end{array}$$

*F* = *eN*_*A*_ is the Faraday. The screen parameter Γ_MSA_ is given by (3)

(94) λP≈(1+βPir)−1[(αλP°HD−ΔλPel)(1+α2β°Pdf)],λP°HD  =(|ZS|Fe6πη〈R〉),ΔλelP=(α|ZS|Fe6πη〈d〉),〈R〉=LLn[g(Rg,LS)],  〈d〉=LSLn[g(d,LS)],d=Rg+12ΓMSA,β°dfP=(23)|ZS|(Rwbs)3(LB〈R〉)[1−ε∞ε]×{(f3−4.808)+|ZS|−16.58}.

Calculations are effectuated with *Fe*/6*π* = 82, *η* = 0.89 cp, *R*_*w*_ = 1.4 Å, *R*_*g*_ = 2.14 ± 0.2, *λ*°_Na^+^_ = 50.1 cm^2^ · *Ω* ^−1^ · eqv_Na^+^_^−1^, *λ* ^°HD^_Chondro_ = 136.1 cm^2^ · *Ω* ^−1^ · eqv_Na^+^_^−1^, 〈*R*〉 = 50.8 Å, *f* = *b*_*S*_/*R*_*g*_ = 2.7 ≥ 2, and *β* ^°df^_*P*_ = 1.47.

Note that the ionic relaxation coefficients *β*_Na^+^_^ir^ and *β*_P_^ir^ are equal [3] and remain approximately constant (~15%) in the studied range of relatively low concentrations, in conformity with the Manning prediction and (8).

Quantitative analysis shows that *β*_*P*_^dfi^ decreases with concentration from 92% to 86%. Therefore, the dielectric friction remains the principal frictional effect in comparison to ionic relaxation effect and electrophoretic effect. On the other hand, the interference effect has a moderation effect of about 25%.

## 7. Conclusion

In order to explain the important negative deviations observed at high dilution in the case of some polyelectrolytes between their experimental conductivities and their theoretical conductivities calculated according to Manning's model or ellipsoidal model, we developed in the present work a more realistic model describing the polyions as chains of successive charged spheres. We have proved that these deviations are due to the dielectric friction effect which remains the principal frictional effect undergone by a stretched polyion even if we take into account the interference effect between the inductions created by the different charged spheres, whereas this effect is nonexistent in the case of Manning's model (infinite thread with a continuous distribution of charge), and it is in general very weak for ellipsoidal polyions. Consequently, it is therefore possible to detect by comparison between conductivity measurements and theoretical results any transition from a coiled configuration (ellipsoidal model) to a stretched configuration (chains of successive charged spheres), during dilution process. We have also underlined the important interdependence between the dielectric friction and the ionic condensation of the counterions, in order to distinguish between the Ostwald regime and the Manning regime for which the degree of condensation is practically constant in a large range ofconcentrations.

## Figures and Tables

**Table 1:**